# Carbon Monoxide Poisoning: From Occupational Health to Emergency Medicine

**DOI:** 10.3390/jcm13092466

**Published:** 2024-04-23

**Authors:** Gabriele Savioli, Nicole Gri, Iride Francesca Ceresa, Andrea Piccioni, Christian Zanza, Yaroslava Longhitano, Giovanni Ricevuti, Maurizio Daccò, Ciro Esposito, Stefano M. Candura

**Affiliations:** 1 Emergency Department, Fondazione IRCCS Policlinico San Matteo, 27100 Pavia, Italy; 2 Niguarda Cancer Center, ASST Grande Ospedale Metropolitano Niguarda, Piazza dell’Ospedale Maggiore, 3, 20162 Milano, Italy; nicole.gri93@gmail.com; 3 Emergency Department and Internal Medicine, Istituti Clinici di Pavia e Vigevano—Gruppo San Donato, 27029 Vigevano, Italy; irideceresa@gmail.com; 4 Department of Emergency Medicine, Polyclinic Agostino Gemelli/IRCCS, Catholic University of the Sacred Heart, 00168 Rome, Italy; andrea.piccioni@policlinicogemelli.it; 5 Geriatric Medicine Residency Program, University of Rome “Tor Vergata”, 00133 Rome, Italy; christian.zanza@live.it; 6 Department of Anesthesiology and Perioperative Medicine, University of Pittsburgh Medical Center (UPMC), Pittsburgh, PA 15260, USA; lon.yaro@gmail.com; 7 Department of Emergency Medicine—Emergency Medicine Residency Program, Humanitas University—Research Hospital, 20089 Rozzano, Italy; 8 Emergency Medicine, School of Pharmacy, University of Pavia, 27100 Pavia, Italy; giovanni.ricevuti@unipv.it; 9 ATS Pavia, Continuità Assistenziale, Via Teodoro Lovati, 45, 27100 Pavia, Italy; maudacc@libero.it; 10 Unit of Nephrology and Dialysis, ICS Maugeri, University of Pavia, 27100 Pavia, Italy; ciro.esposito@unipv.it; 11 Occupational Medicine Unit, Department of Public Health, Experimental and Forensic Sciences, University of Pavia, 27100 Pavia, Italy; 12 Occupational Medicine Unit, Istituti Clinici Scientifici Maugeri IRCCS, 27100 Pavia, Italy

**Keywords:** poison, toxicity, carbon monoxide (CO), emergency department, ED management

## Abstract

Carbon monoxide poisoning remains a leading cause of accidental poisoning worldwide (both at home and at work), and it is also a cause of suicidal poisoning. Such poisoning can arise following prolonged exposure to low levels of CO or following brief exposure to high concentrations of the gas. In fact, despite exposure limits, high safety standards, and the availability of CO alarms, nearly 50,000 people in the United States visit the emergency department each year due to poisoning. Additionally, CO poisoning in the United States causes up to 500 deaths each year. Despite the widespread nature of this form of poisoning, known about for centuries and whose damage mechanisms have been recognized (or rather hypothesized about) since the 1800s, early recognition, especially of late complications, and treatment remain a medical challenge. A well-designed therapeutic diagnostic process is necessary so that indication for hyperbaric or normobaric therapy is correctly made and so that patients are followed up even after acute exposure to diagnose late complications early. Furthermore, it is necessary to consider that in the setting of emergency medicine, CO poisoning can be part of a differential diagnosis along with other more frequent conditions, making its recognition difficult. The last thirty years have been marked by a significant increase in knowledge regarding the toxicity of CO, as well as its functioning and its importance at physiological concentrations in mammalian systems. This review, taking into account the significant progress made in recent years, aims to reconsider the pathogenicity of CO, which is not trivially just poisonous to tissues. A revision of the paradigm, especially as regards treatment and sequelae, appears necessary, and new studies should focus on this new point of view.

## 1. Introduction

Carbon monoxide (CO) intoxication and its severity in oxygen-dependent organisms have been known about since ancient times. To understand its importance, we can think that it has accompanied the history of man since the birth of fire and that of the universe a long time before that [1,2,3,4].

In fact, every time the respiratory tract of a living being comes into contact with smoke from a flame, the manifestations of monoxide poisoning can occur. All of this might appear simple and linear, but CO poisoning often represents an insidious event due to its chemical–physical characteristics and its extraordinary ability to interact with the “queen molecule” of oxygen transport, earning it the name of “silent killer” and “invisible killer” [5].

However, CO is not only this: in recent decades, new studies have emerged that would also suggest its therapeutic potential. However, it is obvious that since the Industrial Revolution, its toxic effects have been the most studied, having dominated for a historically longer period [1,6].

The objective of our research is to review the literature regarding carbon monoxide, both in terms of the symptoms related to CO intoxication and the ways to quickly recognize it in an emergency medicine setting, identifying the signs and symptoms that may arouse suspicion and therefore making a rapid diagnosis. Furthermore, we also focused on the immediate and late consequences, to provide concise knowledge of them, identify them, and, to a certain extent, prevent them.

The ultimate aim of our work is to bring together the pathogenesis, mechanisms of damage, and symptoms related to CO intoxication with a view to its treatment in emergency department settings, providing ideas that can lead to the early recognition of intoxication and its treatment.

## 2. Materials and Methods

We considered any articles in PubMed and Scopus matching specific keywords such as carbon monoxide, carbon monoxide toxicity, carbon monoxide intoxication, CO acute intoxication, CO workplace intoxication, and CO intoxication management, finding 1378 articles. After 3 independent authors and 2 senior experts filtered the selected articles based on emergency medicine, anesthesia, basic medicine, pharmacology, and occupational medicine, we ultimately considered 324 suitable papers.

Finally, 175 papers were analyzed and considered for this review after further screening led to the exclusion of meeting abstracts, books, unavailable manuscripts, original papers without abstracts, brief reports, and papers not in English.

## 3. Results

**Figure 1:**
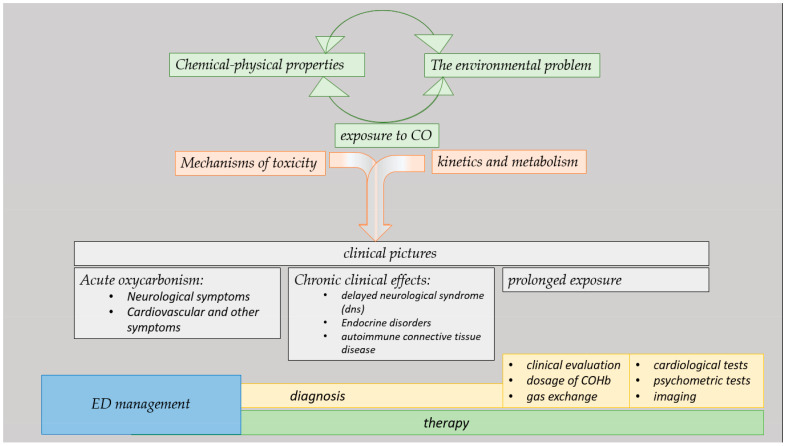
Infographic of results.

The results are schematically represented in Figure 1.

### 3.1. Chemical–Physical Properties and Exposure

Carbon monoxide (CO) is an odorless, colorless, tasteless, non-irritating gas. It is also a highly reactive molecule, flammable, and miscible with air. It is a highly toxic gas primarily produced through the incomplete combustion of organic material and of substances containing carbon (with a lack of oxygen), where it can represent an unwanted byproduct or be methodically produced to exploit its chemical–physical properties (Table 1).

The substances from which CO can be produced include coal, wood, petrol, and diesel. CO can spread and accumulate very insidiously in confined spaces, passing through walls and ceilings (Table 2) [5,6,7,8,9]. 

In working environments, those who carry out their profession in areas with heavy vehicular traffic, such as train drivers, valets, and garage operators, as well as police, firefighters, and kitchen workers, are exposed to CO [10,11]. 

With the identification of its chemical composition at the end of the 19th century, the first experimental studies began with the aim of identifying its mechanisms of toxicity. At the same time, the production of so-called illuminating gas or city gas began (containing 4–10% CO, mixed with other elements, including hydrogen, titanium, and other compounds). As its name suggests, it was used for public and domestic lighting, causing a further increase in cases of poisoning, involuntary or voluntary, such as for suicidal purposes, before being replaced by methane [4,12,13].

The causes of CO exposure and intoxication present variable percentages between different states and communities, being in most cases traceable back to environmental, occupational, or domestic exposure. Causes of home exposure include accidental fires, defective heating systems, and suicidal exposure to vehicle gas. In the workplace, CO poisoning and death should be considered in the presence of machinery located in confined or poorly ventilated spaces [14,15,16,17,18].

### 3.2. The Environmental Problem

CO is not just a work-related issue, as previously described: its role from an environmental point of view is now evident. CO, derived from natural sources (volcanos, combustion, plant metabolism, photochemical oxidation of organic compounds), represents a ubiquitous component of the atmosphere (at a concentration lower than 0.2 ppm). In addition to the CO produced by natural sources, the share produced by anthropogenic sources (motor vehicle traffic, heating systems, industrial emissions, fires, tobacco smoke) must be taken into account. It follows that in large urban centers, where there are areas with high traffic, its concentration reaches considerably higher values (up to 10–20 ppm or more in particularly polluted areas). High concentrations of CO can be easily reached in confined and poorly ventilated areas, such as in the passenger compartments of motor vehicles with malfunctioning exhaust systems, in areas of intense traffic, or in underground garages or garages isolated from the external environment [7].

Exposure can also occur in the presence of stoves, boilers, water heaters, cookers, braziers, and devices installed improperly or in poor maintenance conditions [5,19,20].

Furthermore, CO is also a cause of death in the fishing industry; this should consequently lead to greater control of the equipment on fishing vessels, looking for potential defects. Exposure to CO should also be considered in both commercial and recreational scuba diving, as the molecule can easily accumulate in hoses and equipment [17,18].

The risk of CO poisoning can also be present in environments intended for recreational or sporting activities, such as ice skating rinks, where motorized machinery is used for surface maintenance. CO also contributes to the morbidity and mortality of fire victims due to its chemical ability to interact synergistically with other combustion compounds, such as hydrogen cyanide (HCN), nitrogen and sulfur oxides, ammonia, chlorine, phosgene, halogenated acids, isocyanates, and acrolein, which exert irritating and harmful action in the respiratory system [21,22,23,24].

Tobacco smoke is also a well-known and well-documented source of CO. In smokers, in fact, there is an increase in COHb levels (which can reach 10–12% in heavy smokers). It is certainly true that, in confined spaces, the presence of smokers can cause an increase in the environmental concentration of CO; it is equally true that, however, before the concentration of the gas reaches dangerous levels, irritation due to other components of the smoke becomes intolerable [25].

### 3.3. Kinetics and Metabolism

Although it can be assumed that most CO is absorbed from the environment through the airways, there is a share of CO that is produced through endogenous mechanisms [26,27,28]. 

There is a share of endogenous CO produced by various physiological processes, including heme catabolism and lipid peroxidation. This quota is equal to about 0.42 mL/h and should not produce CO-Hgb levels in excess of 1%. Endogenous CO performs a function similar to that of nitric oxide (NO), which is produced from the amino acid L-arginine. In fact, the two molecules share different biological and chemical properties: the L-arginine–NO system acts as a transduction system in different cellular functions. CO, similar to NO, can perform the same function, binding with the iron atom of the porphyrin group of cytoplasmic guanyl cyclase and with the Fe-S group of various enzymes [7,29,30,31].

Representing a byproduct of heme degradation, it is clear that endogenous CO production increases under pathological conditions that accelerate the metabolism of hemoglobin and other hemoproteins. These disorders include hemolysis, hemolytic anemia, hematoma resorption, thalassemia, Gilbert’s syndrome, and sepsis [7,32,33].

The endogenous formation of CO has been measured using different analytical procedures in different biological systems. In humans, the formation of CO is equal to 0.029 nmol/mg protein/h in the chorionic villi of the term placenta, and it plays a role in its vascular control. The exact mechanism by which CO is produced in the placenta remains unknown; however, it has been postulated that it occurs through a combination of lipid peroxidation and heme degradation on the part of heme oxygenase (HO) [34,35,36].

Furthermore, CO can also be generated by the hepatic metabolism of methylene chloride (dichloromethane: CH_2_Cl_2_). The main CO transport mechanism is respiratory, i.e., through the airways, through which environmental CO is rapidly absorbed. It is transported in the blood in two ways: most of it binds to hemoglobin (its binding to which determines the formation of COHb), while a small amount is dissolved in the plasma. The plasmatic share diffuses into tissues, generating toxic intracellular effects. Another portion (equal to 1–10%) is oxidized into CO_2_ (mainly by mitochondrial cytochrome oxidase). Like absorption, elimination also occurs via the respiratory route. The half-life of CO is 4–5 h [37,38,39].

Due to the endogenous production of CO, normal subjects (non-smokers) have COHb values around 0.5%. This concentration is higher in inhabitants of urban centers or in polluted centers (equal to 1–2%). In smokers and heavy smokers (following inhalation of the CO contained in tobacco smoke), it reaches percentages of up to 8–10% or more. Cigarette smokers are exposed to an estimated 400 to 500 ppm of CO while actively smoking [25,40,41,42].

### 3.4. Mechanisms of Toxicity

In physiological conditions and concentrations, endogenous CO performs various functions, functioning as a neurotransmitter, as a modulator of inflammation, as a regulator of cell proliferation and apoptosis, and as a regulator of mitochondrial function [43,44,45,46].

The main biochemical effects and mechanisms of CO toxicity have been studied and have been known since the 1800s [7,8]. 

Earlier studies attributed the toxic effects of CO to its strong hemoglobin binding capacity; today, this sentence must be presented as a historical note. There is now common agreement that this is its least influential toxic mechanism because tissue toxicity is by far the most influential [13,47,48,49].

CO binds to hemoglobin with an affinity up to 200–250 times greater than that of O_2_, with the consequent formation of COHb, causing a leftward shift in the oxygen–hemoglobin dissociation curve, resulting in tissue hypoxia. However, this affinity has never been proven by studies conducted on humans, only in vitro and animal studies [50,51,52,53,54].

However, decreased peripheral O_2_ release is not the only pathogenetic mechanism; CO poisoning underlies more complex mechanisms of toxicity that go beyond simple COHb formation and oxygen displacement [55,56,57]. 

Carbon monoxide causes oxidative stress (due to increased levels of cytosolic heme) and interrupts cellular respiration (through binding to platelet proteins and cytochrome c oxidase) with the production of reactive oxygen species, which cause apoptosis and neuronal necrosis. The alteration of cellular respiration triggers the mechanisms of response to stress, with the activation of hypoxia-inducible factors and alterations of genetic expression. Furthermore, CO exposure is able to induce inflammation through hypoxia-independent pathways, which also underlie neurological and cardiac damage [58,59,60,61,62,63,64].

The toxic mechanism of CO is determined by a combination of hypoxia and ischemia due to the production of COHb and the direct toxicity that occurs at the cellular level. For this reason, and because of this combination, the severity of clinical effects and symptoms does not correlate directly with the amount of COHb [41,65,66,67,68,69].

In fact, in addition to hemoglobin, CO is also able to interact with other protein structures containing porphyrin groups, including oxidase, catalase, myoglobin (Mb), and various cytochromes, including cytochrome a3 and cytochrome P450. Binding with cytochrome a3 generates a direct toxic effect on cellular respiration, resulting in the formation of oxygen free radicals. In the end, the inactivation of mitochondrial enzymes and the production of free radicals (in particular peroxynitrite) determine the impairment of cellular respiration, with consequent alteration of the electron transport chain. The action exerted on cytochrome a3 is the same as that mediated by cyanide ions [7,70,71,72,73].

On the other hand, binding with Mb determines a reduction in O_2_ transport in the muscles due to the formation of carboxymyoglobin (COMb), with direct effects on the skeletal muscle, inducing toxicity and rhabdomyolysis. 

Myoglobin binding also reduces oxygen availability to the heart muscle, which is a major target (along with the brain) of CO poisoning, resulting in artemia, abnormal contraction, and cardiac dysfunction. In some cases, the onset of compartment syndrome and acute renal failure due to acute tubular necrosis have also been reported [7,25,74,75,76,77,78].

The brain is one of the main targets of the toxic action of CO, representing, together with the myocardium, one of the tissues with the highest oxygen demand. The toxicity of CO at the encephalic level is determined by the activation of guanylate cyclase, with an increase in cyclic guanylyl monophosphate and consequent cerebral vasodilatation. In animal models of CO poisoning, cerebral vasodilation has ultimately led to a loss of consciousness (Table 3) [25,79,80,81].

### 3.5. Clinical Pictures

CO poisoning is a common and potentially life-threatening intoxication. It is characterized by an extremely variable inter-individual symptom train, often non-specific symptoms, and late neurological sequelae. The symptoms depend on the duration of exposure and CO levels [25,82].

The clinical pictures can be divided into four categories: acute oxycarbonism, chronic clinical effects, delayed clinical effects, and the effects of prolonged exposure. Furthermore, a paragraph will be devoted to the delayed neurological syndrome, which must be carefully suspected in the presence of neurological symptoms and in the presence of a positive history of CO poisoning [83,84].

Most intoxications occur during the winter period, involving the economically disadvantaged strata of the population living in apartments without centralized heating systems. Often, the symptomatology involves several people belonging to the same family. The presence of these factors and the presence of collective symptoms must raise suspicion of CO poisoning [5,19].

### 3.6. Acute Oxycarbonism

The picture of acute (or subacute) CO intoxication is characterized primarily by neurological and cardiological symptoms and signs; the severity of intoxication is correlated both with the environmental concentration of CO and with the duration of exposure. The heart and the brain are the organs that show the first signs of injury, being the tissues most dependent on an oxygen supply. In the following two paragraphs, the neurological symptoms, cardiological symptoms, and other symptoms of CO poisoning will be analyzed.

#### 3.6.1. Neurological Symptoms 

The initial CO poisoning symptoms include headache, nausea, and dizziness. With increasing exposure, the symptoms become more severe and characteristic. In healthy subjects, on average, the first symptoms appear after 4 h of exposure to 200 ppm [5,25,41,85,86].

Headache is the earliest and most common symptom; it has the characteristics of being frontal and continuous. In the case of neurological symptoms, imaging techniques (CT, MRI) often document degenerative symmetrical alterations, with the involvement of the nuclei of the base and of the subcortical white matter. These alterations rarely involve other areas of the brain. The presence of these alterations has a negative prognostic significance. Increased exposure results in other neurological symptoms, such as altered mental status, confusion, syncope, and convulsions, up to acute syndromes that mimic stroke and coma. Isolated seizures are very common in pediatric patients, with other neurological symptoms appearing more subtle [25,41,87,88,89,90,91].

Furthermore, the presence of systemic hypotension during CO poisoning is directly related to the severity of the structural damage at the level of the central nervous system. In particular, experiments conducted on resus monkeys, which had been exposed for 75–325 min and had received from 0.1% to 0.3% CO, showed the onset of various effects on the central nervous system, including severe deficits, paralysis of the limbs, alterations of muscle tone, deafness, and blindness. The characteristic brain lesion was bilateral destructive leukoencephalopathy, with major involvement of the frontal and posterior parietal regions. Lesions of the globus pallidus and hippocampus were also noted. The size of the lesions correlated not only with hypotension but also with the degree of metabolic acidosis. Surprisingly, injury severity was not correlated with the extent of hypoxia, suggesting that it was crucially determined by other factors, although hypoxia was a preconditioning factor [92,93,94].

#### 3.6.2. Cardiovascular and Other Symptoms

The primary cardiovascular effect of CO poisoning is hypoxia-induced tachycardia. Subsequently, more significant exposures determine the onset of hypotension, arrhythmia, ischemia, infarction, and, in extreme cases, cardiac arrest. Cardiac arrhythmia represents the main cause of early death after exposure to monoxide. Hypotension is determined by a combination of direct cardiac damage (due to hypoxia/ischemia), myocardial depressant activity (due to the action of myoglobin), and peripheral vasodilatation. Furthermore, CO can exacerbate underlying cardiovascular diseases, making this group of patients particularly fragile and particularly susceptible to the onset of cardiovascular symptoms [57,62,95,96,97,98,99].

CO poisoning can cause rhabdomyolysis and acute renal failure, as an effect and as a consequence of direct toxic damage to the skeletal muscle and the ability of CO to bind myoglobin [64,100,101].

Less common symptoms and signs include skin blisters and non-cardiogenic pulmonary edema. Contrary to popular belief, a “cherry red” skin color, which is considered a hallmark of CO intoxication, is not commonly observed in clinical practice [102,103].

Then, there are alterations in the acid–base balance, which vary according to the severity of the intoxication. In grade 3 intoxications (medium-severity), the most common acid–base disorder is respiratory alkalosis, to compensate for hypoxia; in grade 4 (more severe cases), the most frequent disorder is instead metabolic acidosis (due to the overproduction of lactates resulting from hypoxia). In the latter case, the skin being a bright red color (cherry red), induced by vasodilation, and the presence of COHb are often associated. Possible complications include acute pulmonary edema, aspiration pneumonia, rhabdomyolysis, compartment syndrome, and renal failure. COHb concentrations >50–70% are often fatal (Table 4) [5,19,25,104,105,106].

Pregnant women are particularly fragile to exposure to CO, where intoxication represents a unique scenario, as CO easily crosses the placenta. Maternal exposure to CO would result in higher peak fetal COHb levels, with slower elimination of CO than that of maternal COHb. Severe maternal exposure in humans is linked to an increased risk of miscarriage, stillbirth, anatomical malformations, and neurological disability. Maternal symptoms do not correlate directly with the severity of fetal symptoms: even in slightly symptomatic mothers, the effects on the fetus can be serious, up to fetal death. The fetus being of an early gestational age is more closely related to anatomical malformations; on the other hand, functional disturbances and poor neurological development can be evident at any gestational age. Fetal damage is more evident in the brain, particularly in the basal ganglia and globus pallidus at autopsy. If the infant reaches full term, it often has a low birth weight and may develop postnatal neuropsychological developmental delay [41,107,108,109,110,111,112,113].

Because of CO’s tighter binding to fetal than adult hemoglobin, infants are more vulnerable to its effects, making carbon monoxide poisoning potentially lethal in newborns. Pediatric patients are also more vulnerable to the effects of CO due to a higher oxygen uptake rate and higher metabolic rate. Despite this, the symptomatological set of pediatric patients is often non-specific (nausea and vomiting), causing difficulties in differential diagnosis, as this mimics viral disease [114,115,116,117].

COHb levels do not correlate with the severity of the clinical picture; this happens only for values greater than 40–50%. This depends on several factors. First, the COHb concentration does not reflect the amount of gas actually inhaled, as it decreases by half every 4–5 h (more rapidly if oxygen is administered). Second, COHb is not directly representative of the amount of CO bound to intracellular targets (myoglobin, cytochromes), which contribute significantly to its toxicity. Third, it is necessary to take into account factors such as individual sensitivity and the presence of concomitant pathologies. In intoxicated people who survive to receive assistance in hospital, the mortality is around 3%. At autopsy, the cherry red color of the blood and hypostasis in the muscles and organs are characteristic; the lungs are distended and edematous, while the brain and myocardium may show areas of ischemic necrosis [5,19,25,89].

### 3.7. Presentation and Management in EDs

Patients can therefore arrive in EDs with all these acute symptoms, which must be placed in differential diagnosis with many other pathologies. Cardiac rhythm disorders can be caused by other toxicants, metabolic disorders, ischemic disorders, and many other things. Chest pain can be caused, among other emergencies, by cardiac ischemia, myocarditis, diseases of the thoracic aorta, pulmonary embolism, pneumothorax, pneumomediastinum, diseases of the pleura and esophagus, and many other things [118].

Neurological symptoms can be an expression of other emergencies, such as cerebral ischemic pathology, Acute Disseminated Encephalomyelitis (ADEM), cerebral hemorrhage, neoplastic masses, and metabolic or infectious diseases [118,119].

All these pathologies are part of a differential diagnosis and are emergencies that require timely exclusion/confirmation diagnoses and immediate treatments. Obviously, pathologies that do not represent emergencies are also part of the galaxy of differential diagnosis, as in the case of syncope due to benign causes. Conversely, patients who experience syncope due to carbon monoxide intoxication may suffer head trauma or other trauma, with the consequent activation of other therapeutic diagnostic pathways, perhaps also due to associated therapies that increase the risk of traumatic bleeding and therefore require their activation [120,121]. 

The activation of these protocols must not delay the diagnosis of carbon monoxide poisoning. It should be remembered that a fall at home can cause serious trauma to an elderly person, resulting in worse outcomes [122].

Patients arriving accompanied by local services may already be suspected to have monoxide poisoning if a CO detector has alerted the rescuers. However, this may not be the case because the subject may no longer be in the place, however nearby it is, where intoxication occurred. Patients who arrive in the ED, however, represent a more complex diagnostic challenge because it may not be possible to collect a reliable medical history, as demonstrated in elderly patients or patients with mental disorders or trauma [123,124,125].

In EDs, a differential diagnosis might be challenging due to the lack of an accurate medical history and information about the patient’s baseline cognitive function, especially when caregivers are not available. For example, up to 40% of older adults are unaccompanied, especially when patients arrive by ambulance and/or from long-term care facilities [126,127].

The difficulties listed herein increase in conditions of ED crowding, a condition that is increasingly more frequent and related to worse outcomes [128,129].

In conditions of crowding, the doctor–patient and nurse–patient relationships become unbalanced, making the process more difficult and more susceptible to worse outcomes, from perceived quality to mortality [130,131].

Since the therapeutic diagnostic process begins at triage, it is necessary to remember how triage, in real life, is a complex process and how crowding influences the triage itself [132].

In the opinion of the authors, performing an arterial or venous blood gas analysis in triage for all neurological disorders, cardiac rhythm disorders, and chest pain is considered the proper path. In fact, a good blood gas analysis allows you to detect not only the presence of CO but also hypoglycemia, low hemoglobin values (which could cause, for example, discrepancy angina), and other alterations and therefore provides elements for better assessment of a patient. In situations where blood gas analyses cannot be performed in triage, it is advisable for them to take place at first medical contact. The authors recommend good collaboration with the relevant anti-poison center, either in each individual case or with training and refresher courses in selected cases. Depending on the severity of the symptoms and the characteristics of the patient, hyperbaric chamber therapy may be necessary, as will be illustrated in detail below in the description of therapy. For this to happen smoothly, the regional hub-and-spoke system must be well defined, and the dialogue between structures must be constant. Patients, whether they do not require hyperbaric therapy or are waiting for or returning from hyperbaric therapy, can benefit from observation and therapy in dedicated areas such as holding areas or observation areas with a medium–high intensity of care within EDs. These areas can contribute to improving outcomes and adherence to guidelines and determining a better appropriateness of treatments and hospitalizations, as already demonstrated in various pathologies and cases of fragile patients [133,134,135].

### 3.8. Chronic Clinical Effects

CO’s chronic clinical effects are less well known due to the inherent difficulties in quantifying the degree of CO exposure compared to the degree of neurological impairment. On exposure to low levels of CO, the onset of a neurological syndrome characterized by headache, dizziness, nausea, cerebellar dysfunction, and cognitive and mood disturbances has been reported; this symptomatology tends to regress and is revealed once the subject is removed from a poisonous environment. However, these studies have confounding factors, as exposure data are lacking. Chronic exposure to CO has also been associated with decreased physical performance, exacerbation of heart disease, and low birth weight. Furthermore, chronic hypoxia determines polycythemia and cardiomegaly [136,137,138,139,140,141].

### 3.9. Delayed Neurological Syndrome (DNS)

Delayed neurological syndrome (DNS), or delayed encephalopathy, is characterized by a neurological clinical picture that arises after acute CO intoxication and after a period of apparent recovery. It generally occurs within 40 days of exposure, although longer latencies, up to 8 months, have also been reported. The onset of the syndrome is unpredictable. The true prevalence of DNS is difficult to determine; the estimates range from 1% to 47% of patients after CO poisoning. The exact incidence rate is also unclear. Studies using rigorous methodologies, including neuropsychological testing, report the frequency to be as high as 67%. As for its pathogenesis, it is not clear. In addition to the CO toxicity mechanisms described in the previous paragraphs, peroxidation of the lipids in the brain with free radical damage and overstimulation of the excitatory amino acid receptors would also be implicated. Oxygen therapy may have a preventive role, counteracting these mechanisms of damage [83,84,142,143,144].

The risk factors include an age over 40 years, cardiovascular disease, prolonged exposure to CO (greater than 1 h), electroencephalographic abnormalities, and coma (especially if prolonged and followed by persistent asthenia and vertigo). It has been observed that patients with a broader symptomatic clinical picture at onset have a greater chance of developing long-term neurological sequelae [140,145,146,147].

The clinical symptoms of DNS overlap with those of any known neurologic clinical syndrome with neurologic or psychiatric symptoms, possibly including motility disorders (parkinsonism, choreoathetosis), gait disturbance, autonomic dysfunction resulting in incontinence (urinary and fecal), seizures, and cortical blindness. Symptoms similar to those of multiple sclerosis, peripheral neuropathies, Wernicke’s aphasia, and Korsakoff syndrome, but also agnosia, mutism, dementia, personality changes, psychosis, and bipolar syndromes, have also been reported. Furthermore, the use of neuropsychological tests allows us to demonstrate more subtle alterations, which are not evident on neurological examination. The syndrome can resolve spontaneously, but healing can take more than two years. In other cases, DNS can result in permanent neurological damage, resulting in severe impairment of a patient’s quality of life [73,145,146,148].

### 3.10. Endocrine Disorders

As has been previously reported, the heart and brain represent the organs most affected by carbon-monoxide-induced hypoxia due to their high metabolism [149,150,151,152]. 

The effects of CO on the heart and neurological sequelae are among the main causes of mortality following CO intoxication. In addition to hypoxia, recent evidence has suggested that CO intoxication can induce inflammatory and immunological reactions in several organs, resulting in endocrine disorders. These endocrine disorders are induced at the cerebral level through the alteration of the hypothalamic–pituitary axis by carbon monoxide intoxication, which has repercussions at the peripheral level for the endocrine organs subjected to the control of the axis. In particular, we can cite a study conducted in Taiwan (in 2017), where it was found that the risk of developing diabetes increased by approximately 2 times after carbon monoxide intoxication due to a combination of damage to the hypothalamus, brainstem, and pancreas. The risk of diabetes in the presence of CO intoxication increases in patients <35 years of age and in elderly patients (≥65 years of age), of the female sex, and with hyperthyroidism, heart failure, or polycystic ovary syndrome. This risk lasts up to 4 years after exposure [153,154,155].

CO intoxication also leads to alterations in the adrenal glands, responsible for the production of three classes of hormones fundamental to the correct functioning of important functions in our organism (glucocorticoids, mineralocorticoids, and androgens). In particular, control of cortisol secretion (the most important glucocorticoid produced by the adrenal glands) is strictly regulated by the hypothalamic–pituitary–adrenal axis. Any dysfunction of this important pathway (centrally or peripherally), including that caused by CO, can contribute to the onset of adrenal insufficiency [151,156,157]. Furthermore, the risk of developing adrenal insufficiency is greater in patients who experience acute respiratory failure (ARF), in women and younger populations, and a year after follow-up [152].

Another endocrine disorder caused by CO intoxication is hypothyroidism. Similarly to adrenal insufficiency, hypoxia and the consequent production of free radicals, resulting in an inflammatory substrate, can one of the causes that determines the alteration of thyroid function and then hypothyroidism. Thyroid function and the secretion of thyroid hormones are regulated by the hypothalamic–pituitary–thyroid (HPT) axis. Hypothyroidism can therefore be caused both by lesions at the central level (hypothalamus and pituitary gland) and in the local organ (thyroid gland) [158,159,160].

### 3.11. Autoimmune Connective Tissue Disease

Although the evidence and studies on the matter are limited, CO intoxication can lead to an increased risk of developing autoimmune connective tissue diseases.

Connective tissue diseases include a spectrum of pathologies (systemic lupus erythematosus, rheumatoid arthritis, scleroderma, Sjögren’s syndrome, mixed connective tissue disease), characterized by spontaneous hyperactivity of the immune system (with consequent overproduction of antibodies), to which both genetic and environmental factors contribute. Carbon-monoxide-induced hypoxia, oxidative stress, and inflammation can increase the risk of autoimmune disease by mediating the production of autoantibodies, leading to T cell dysfunction and oxidative modification of self-antigens. Oxidative stress, on the other hand, would be implicated in the pathogenesis and mechanism of damage of systemic lupus erythematosus. As regards Sjøgren’s syndrome, this would be triggered by oxidative stress and mitochondrial dysfunction [160,161,162,163,164,165].

In a recent study, participants with CO poisoning were found to have a higher risk of autoimmune connective tissue disease than those who had not been poisoned, after adjusting for sex, diabetes mellitus, infectious diseases (H. zoster, HIV, Lyme disease, hepatitis, mononucleosis), liver disease, malignancy, hypertension, hyperlipidemia, coronary heart disease, congestive heart failure, chronic obstructive pulmonary disease, and drug abuse. This increased risk is observed even after 4 years of follow-up; therefore, patients who experience CO intoxication should undergo a long follow-up, aiming to highlight the onset of connective tissue diseases and other autoimmune disorders [163,164,165,166,167].

### 3.12. Effects of Prolonged Exposure

Chronic exposure to CO remains a controversial topic, as the possibility of its occurrence is very doubtful. In reality, it is believed that the clinical picture that occurs, defined as chronic oxycarbonism, is more correlated with the sequelae of repeated (and misunderstood) episodes of subacute intoxication. This syndrome has been described in workers exposed to CO for years, characterized by a triad of symptoms (asthenia, headache, dizziness). This symptomatology can also be associated with both neurological (parkinsonism, epilepsy, otovestibular disorders) and cardiac (arrhythmia, precordial pain) manifestations [168,169,170,171].

A controversial aspect, not yet fully clarified, would concern the possibility of CO acting as an atherogenic trigger. On the one hand, although many studies tend to exclude the role of CO in cardiovascular pathology, on the other hand, studies conducted on various animal species would demonstrate that repeated exposure to relatively low doses (such as to determine COHb levels of 5–10%) would favor the appearance of aortosclerosis, above all in the coronary artery. In agreement with the latter observations, several epidemiological studies document a higher rate of mortality from ischemic heart disease in subjects occupationally exposed to CO, including workers exposed to motor vehicle emissions. The atherogenic mechanism of CO may be related to endothelial hypoxic insult, which would partially account for the increased cardiovascular risk in smokers [10,11,41,168,169,170,171,172].

### 3.13. Diagnosis

#### 3.13.1. Clinical Evaluation

Patient evaluation should carefully evaluate the adequacy of ventilation and perfusion, exposure history, neurologic examination, and cardiac evaluation. The diagnosis of acute CO poisoning is based, when possible, on anamnesis (recent exposure), the clinical picture (signs and symptoms), and any evidence of collective symptoms. 

#### 3.13.2. Dosage of COHb: The Fundamental Diagnostic Test

The dosage of COHb represents the fundamental diagnostic test: levels > 3–5% (>10% in smokers) are indicative of intoxication. To evaluate COHb levels, the broad consensus is to perform a venous blood gas analysis. In fact, given the high diffusibility of CO, the arterial and venous vascular compartments are in rapid equilibrium, and venous sampling is therefore sufficient to determine CO. Note that COHb levels are absolutely not an expression of the severity of intoxication; they are in fact an indicator of exposure, but over time, they normalize. There are pulse oximeters with CO detection, which are particularly useful, at least for a first evaluation in children, for whom it could be difficult or traumatizing to take a blood sample. The diagnosis of CO intoxication includes several parameters and must be carefully investigated due to its non-specific symptoms. The basis of the diagnosis is the measurement of the percentage of COHb; this value is highlighted through blood gas analysis of the arterial blood. An invasive measurement of carboxyhemoglobin remains the diagnostic standard; on the other hand, the main disadvantage of this technique is its unavailability in an extra-hospital context. Therefore, COHb levels remain the only reliable factor for a diagnosis of CO intoxication. Alternatively, CO oximetry can also be performed in a non-invasive manner. 

Already from studies conducted in 1987, it emerged that the main problem in the diagnosis of CO intoxication is determined by the fact that measuring the peripheral oxygen saturation using a standard pulse oximeter produces false values. This is due to the fact that most pulse oximeters only measure two wavelengths of light. Regular pulse oximeters do not detect the difference between oxyhemoglobin and carboxyhemoglobin and therefore identify carboxyhemoglobin as oxyhemoglobin. For example, even with carboxyhemoglobin levels of 70%, a pulse oximeter shows oxygen saturation values of 92%, demonstrating that the pulse oximeter reads carboxyhemoglobin as if it were composed of 90% oxyhemoglobin and 10% reduced hemoglobin. It therefore appears essential to have a device capable of diagnosing CO poisoning in a non-invasive way, so as to confirm the presence of carbon monoxide poisoning on site in the presence of suspicion.

The use of a pulse oximeter capable of detecting CO and providing an estimate of these data is useful in quickly highlighting the presence of intoxication. They, unlike regular pulse oximeters, use eight wavelengths of light, measuring multiple different types of human hemoglobin. These devices are therefore able to distinguish between oxyhemoglobin, carboxyhemoglobin, and methemoglobin. In conclusion, non-invasive COHb analysis using pulse CO oximetry represents an easy-to-handle method to facilitate the diagnosis of CO intoxication. It is necessary to remember that this diagnosis, as previously reiterated, must be supported by further investigations, including objective examination of the patient and radiological diagnostics.

#### 3.13.3. Arterial Hemogram Analysis: Evaluation of Gas Exchange

Arterial hemogram analysis then provides important information on the adequacy of gas exchange and on metabolic acidosis, as well as on carboxyhemoglobinemia; it should therefore be performed when clinically indicated. For severe intoxication and in intubated patients, it is essential to carry out arterial blood gas analysis, where base excess can be used as a predictive index for acute mortality and morbidity (especially post-anoxic encephalopathy). 

#### 3.13.4. The Role of Blood Chemistry Tests 

There is a broad consensus in the literature that specific blood chemistry tests are not necessary. Undoubtedly, the anamnesis and clinical state will guide practitioners in terms of the need to perform myoglobinemia, CPK-MB, and TNI testing, remembering that long exposures to CO and immobility for many hours can lead to very high myoglobinemia values. 

#### 3.13.5. Recommended Cardiological Tests

It is unanimously recommended to perform an ECG before hyperbaric treatment due to both the arrhythmogenic and ischemic effects of intoxication, regardless of age. Carbon monoxide poisoning can exacerbate angina and cause heart damage, even in people with undamaged coronary arteries. Therefore, poisoned patients must undergo a cardiovascular investigation, including electrocardiography and measurement of cardiac enzymes. If these detect myocardial damage, cardiological tests should be requested. Additionally, diagnostic color Doppler echocardiography and coronary angiography are recommended for patients in whom signs of cardiac ischemia persist. Recent studies support the use of new biochemical indicators such as B-type natriuretic peptide (BNP) for the identification of the cardiotoxicity of early CO poisoning. However, studies on this in the literature are very limited, and the dosage of the enzyme should be considered for research purposes. 

#### 3.13.6. Psychometric Tests: Post-Interval Syndrome

Acute psychometric tests are useful, above all to investigate any onset of post-interval syndrome (a complication of CO poisoning characterized by neuropsychiatric symptoms, of varying severity, and with a potentially delayed onset after a free interval of 3 weeks). It is necessary to point out the difficulty of administering these tests to numerous people in emergency situations, such as in the middle of the night, where medical and nursing staff are naturally limited. In this regard, other difficulties highlighted are linguistic barriers, i.e., the need to have tests in multiple languages (Arabic, Romanian, Albanian, Chinese, etc.) and therefore the possibility of interpreting them. In fact, in Italian case studies, approximately 35% of patients intoxicated by CO and treated at our center are not Italian speakers. 

#### 3.13.7. The Role of Brain Imaging

However, it is not recommended nor is it considered appropriate to perform a brain CT scan before hyperbaric treatment if the diagnosis is certain, unless the patient shows clear signs of head trauma following a fall due to intoxication. A chest X-ray is essential if the patient is subjected to mechanical ventilation, if it is not possible to reconstruct a reliable medical history, or if data emerge relating to previous episodes of spontaneous pneumothorax. Magnetic resonance imaging (MRI) of the brain is usually performed for research purposes or, in rare cases, for clinical indications, such as to exclude disorders unrelated to exposure to carbon monoxide. MRI has been shown to reveal abnormal findings after carbon monoxide poisoning. In cases of intentional carbon monoxide poisoning, clinical judgment, psychiatric evaluation, and further toxicological laboratory investigations are necessary for the detection of alcohol, benzodiazepines, narcotics, amphetamines, or other such agents. It is clear that in severely intoxicated patients (for example, patients undergoing mechanical ventilation), the possibility of the coexistence of aspiration pneumonia will extend the range of tests required. 

#### 3.13.8. Differential Diagnosis and the Evaluation of Associated Intoxications

Since the symptoms of CO intoxication are highly variable, and since CO is defined as “the great imitator”, differential diagnosis can be difficult. The clinical picture can easily be mistaken for manifestations of headache, gastroenteritis or flu syndrome, psychiatric disorders, hypoglycemia, other intoxications (food, drugs, alcohol, organic solvents), or with more serious cerebrovascular disease, acute or chronic. In these cases, a correct diagnosis is not only necessary (obviously) to start the correct therapy but also to avoid sending an intoxicated person back into a contaminated environment [19,105,169,171,172]. We assert that it is necessary to also evaluate the presence of other combustion compounds, such as hydrogen cyanide (HCN), nitrogen and sulfur oxides, ammonia, chlorine, phosgene, halogenated acids, isocyanates, and acrolein.

### 3.14. Therapy

In cases of acute intoxication, the first measure is represented by the removal of the patient from the contaminated environment as quickly as possible. If necessary, first aid resuscitative maneuvers should be applied. Even the rescuers must be minimally exposed to the risk of intoxication, with them wearing suitable respiratory protective equipment to avoid chain injuries. The risk of explosions must be mitigated by not introducing lights or flames into the polluted environment.

The antidote to CO is oxygen, which must be administered in pure form (100%). There is broad agreement that the first treatment is the administration of normobaric oxygen to intoxicated patients, either with high-flow oxygen or with 100% oxygen if the carboxyhemoglobin values are less than 5% since the administration of normobaric oxygen accelerates CO elimination. The use of O_2_ allows for the half-life of COHb to be lowered to about one hour. It is necessary to mention that this has not been verified by some studies, which have not shown a significant reduction in neurological sequelae following the administration of normobaric oxygen. However, its administration is justified by its safety, ready availability, and low cost. Treatment with high-flow normobaric oxygen therapy, using a one-way valve device, must be started from the time of first aid and continued until the patient enters a hyperbaric chamber if this treatment is deemed appropriate [19,41,55,88,89,173,174,175,176].

Regarding the rationale of using hyperbaric oxygen therapy (HBOT) for CO poisoning, as well as to accelerate the dissociation of CO from hemoglobin, it has other effects. In experiments conducted on animals poisoned by CO, it was shown that the use of HBOT not only reduces the binding of CO to hemoglobin but also reduces the binding of CO to other heme-containing proteins (for example, cytochrome a3) that influence cellular metabolism. Furthermore, HBOT alters neutrophils’ mechanisms of adhesion to the endothelium; decreases free-radical-mediated oxidative damage; reduces neurological deficits and overall mortality when compared with normobaric oxygen therapy (NBO); improves arterial and tissue oxygen tension, facilitating the elimination of CO; increases the production of adenosine triphosphate; and reduces oxidative stress and inflammation. In addition, it seems that the use of hyperbaric oxygen reduces the rate of cognitive sequelae at 12 months [41,177,178,179,180,181,182,183,184,185,186,187].

The Undersea and Hyperbaric Medical Society recommends hyperbaric oxygen therapy for patients with severe CO poisoning, exhibiting symptoms such as transient or prolonged unconsciousness, neurological abnormalities, cardiovascular dysfunction, or severe acidosis. Patients older than 36 or who have been exposed for 24 h or more (including intermittent exposures) or with carboxyhemoglobin levels of 25% or higher should also receive hyperbaric oxygen therapy (Table 5) [178,179,180,181,182,183,184,185,186,187,188,189].

Children, pregnant women, and adult patients—regardless of their carboxyhemoglobin values—who have cardiac or neurological toxicity must be transported to specialized centers equipped with a hyperbaric chamber: in fact, at 2.5 atmospheres absolute, COHb’s half-life is reduced to 20 min [177,178,179,180,181].

Oxygen therapy must also be accompanied by symptomatic therapy, with possible correction of the acid–base balance, and intensive supportive care, including airway management, blood pressure support, and stabilization of cardiovascular status. As far as the follow-up is concerned, it lasts for six months. Readmission to work is subject to careful assessment of any neurological and cardiac sequelae [67,68,69,145,190,191,192].

Patients with carbon monoxide poisoning should be followed up clinically after discharge. The extent and rate of recovery after poisoning is variable, and recovery is often complicated by the development of sequelae, which may persist after exposure or develop weeks after poisoning and be permanent.

Unfortunately, there is no specific therapy available for the sequelae of carbon monoxide poisoning.

## 4. Conclusions

Carbon monoxide poisoning remains a difficult challenge for emergency doctors due to its insidious clinical and clinical course. The correct patient assessment, particularly the administration of cognitive tests, is time-consuming and cannot always be easily performed today in our increasingly crowded EDs. Effective multi-professional and multidisciplinary coordination between various emergency medicine figures, such as triage nurses, the emergency doctor of the receiving hospital, the poison control center, and the doctors of HUB centers for hyperbaric therapy, is necessary for efficient treatment. This coordination must be expressed according to a therapeutic diagnostic path that allows patients to be followed up over time by dedicated centers to diagnose delayed onset sequelae in good time.

## Figures and Tables

**Table 1:** Characteristics of carbon monoxide.

CAS 630-08-0 EC: 006-001-00-2 Other names: Carbon monoxide, carbonic oxide	Molecular formula: CO
Physical state at 25 °C: Gas (odorless and tasteless) Melting point: −205 °C Boiling temperature: −191 °C	Molecular weight: 28.01 g/mol Vapor pressure: Not applicable
Relative vapor density (air = 1): 0.97	Solubility in water: 2.3 mL/100 mL at 20 °C
Self-ignition temperature: 605 °C Flash point: −101.6 °C Explosive limit: 12.5–74.2 vol (%)	Conversion factors: - 1 mL/m^3^ = 1.83 mg/m^3^ - 1 ppm = 1.14 mg/m^3^ - 1 mg/m^3^ = 0.87 ppm

**Table 2:** Main industrial activities with exposure to CO.

Industrial Sector	Processing
Fuel	- Treatment of coke oven gas - Coke extinguishing - Production and distribution of illuminating gas - Gasification of solid fuels
Siliceous products	- Use of ovens
Chemistry and petrochemistry	- Use of ovens - Production of CO and its mixtures - Production of calcium carbide (CaC_2_), acetylene (C_2_H_2_), hydrogen cyanide (HCN), and organic compounds
Metallurgy and engineering	- Use of ovens - Welding and cutting with a blowtorch - Repair, maintenance, and use of engines

**Table 3:** CO toxicity mechanisms.

EFFECT	MECHANISM
HEMOGLOBIN ❖ Decreased oxygen-carrying capacity ❖ Less oxygen transfer to the tissues	❖ Formation of carboxyhemoglobin (COHb) ❖ Haldane effect
TISSUES ❖ Decreased muscular oxygen availability ❖ Lower cellular utilization of oxygen ❖ Slowing down of metabolic functions	❖ Inhibition of mitochondrial respiration ❖ Enzyme inhibition ❖ Cytochrome P450 inhibition

**Table 4:** Classification of CO intoxication according to the Italian SIMEU guidelines.

SEVERITY CLASS	SIGNS AND SYMPTOMS
**ASYMPTOMATIC** (grade 1)	Absent (with positive COHb values)
**MILD** (grade 2)	Headache, dizziness, nausea, vomiting
**AVERAGE** (grade 3)	Mental confusion, slow thinking, blurred vision, asthenia, ataxia, abnormal behavior, shallow breathing, exertional dyspnea, tachypnea, tachycardia, abnormal psychometric test
**SERIOUS** (grade 4)	Drowsiness, sensory blunting, coma, convulsions, syncope, disorientation, brain CT changes, hypotension, chest pain, palpitations, arrhythmias, ECG ischemic signs, pulmonary edema, myonecrosis, skin bullae, lactic acidosis

**Table 5:** Indications for hyperbaric therapy.

INDICATIONS FOR HYPERBARIC THERAPY
Syncope
Coma
Convulsions
Altered mental state
Cerebellar signs
Carboxyhemoglobin > 25%
Fetal distress (in pregnancy)

## Data Availability

Not applicable.

## References

[1] Megas I.F., Beier J.P., Grieb G. (2021). The History of Carbon Monoxide Intoxication. Medicina.

[2] Ehrenfreund P., Spaans M., Holm N.G. (2011). The evolution of organic matter in space. Philos. Trans. R. Soc. A Math. Phys. Eng. Sci..

[3] Abel T., Bryan G.L., Norman M.L. (2002). The Formation of the first star in the universe. Science.

[4] Cruickshank W. (1801). Some observations on different hydrocarbonates and combinations of carbone with oxygen, ect. In reply to some of Dr. Priestley’s late objections to the new system of chemistry. J. Nat. Philos. Chem. Arts.

[5] Petrolini V., Bigi S., Vecchio S., Lonati D., Giampreti A., Locatelli C., Butera R., Manzo L. (2008). Il monossido di carbonio: “killer silenzioso” e “grande imitatore”. Emerg. Care J..

[6] Kim H.H., Choi S. (2018). Therapeutic Aspects of Carbon Monoxide in Cardiovascular Disease. Int. J. Mol. Sci..

[7] ATSDR, Agency for Toxic Substances and Disease Registry (2012). Toxicological Profile for Carbon Monoxide.

[8] Candura F., Candura S.M. (2002). Elementi di Tecnologia Industriale a Uso Dei Cultori di Medicina Del Lavoro.

[9] Chenoweth J.A., Albertson T.E., Greer M.R. (2021). Carbon Monoxide Poisoning. Crit. Care Clin..

[10] Candura S.M., Verni P., Minelli C.M., Rosso G.L., Cappelli M.I., Strambi S., Martellosio V. (2006). Rischi professionali nelle Forze dell’Ordine. G. Ital. Med. Lav. Erg..

[11] Sancini A., Tomei F.T.G., Caciari T., Di Giorgio V., Andrè J.C., Palermmo P., Andreozzi G., Nardone N., Schifano M.P., Fiaschetti M. (2012). Urban pollution. G. Ital. Med. Lav. Erg..

[12] Bernard C. (1857). Leçon Sur Les Effects des Substances Toxiques et Medicamenteuses. https://gallica.bnf.fr/ark:/12148/bpt6k773289.texteImage.

[13] Haldane J.S. (1895). The reation of the action of catabolic oxide to O_2_ tension. J. Physiol..

[14] Chang S.S., Chen Y.Y., Yip P.S.F., Lee W.J., Hagihara A., Gunnell D. (2014). Regional changes in charcoal-burning suicide rates in East/South East Asia from 1995 to 2011: A time trend analysis. PLoS Med..

[15] Valent F., McGwin G., Bovenzi M., Barbone F. (2002). Fatal work-related inhalation of harmful substances in the United States. Chest.

[16] Byard R.W. (2013). Commercial fishing industry deaths—Forensic issues. J. Forensic Leg. Med..

[17] McDermott J.H., Reynard C., Perry J., Dear J.W., Child F., Jenner R. (2018). Acute carbon monoxide toxicity in a paediatric cohort: Analysis of 10 boys poisoned during a scuba diving lesson. Clin. Toxicol..

[18] Allen H. (1992). Carbon monoxide poisoning in a diver. Arch. Emerg. Med. J..

[19] Tomaszewski C., Flomenbaum N.E., Howland M.A., Goldfrank L.R., Lewin N.A., Hoffman R.S., Nelson L.S. (2006). Carbon monoxide. Goldfrank’s Toxicologic Emergencies.

[20] Kales S.N. (1993). Carbon monoxide intoxication. Am. Fam. Physician.

[21] Pelham T.W., Holt L.E., Moss M.A. (2002). Exposure to carbon monoxide and nitrogen dioxide in enclosed ice arenas. Occup. Environ. Med..

[22] Locatelli C., Candura S.M., Maccarini D., Butera R., Manzo L. (1994). Carbon monoxide poisoning in fire victims. Indoor Environ..

[23] Anseeuw K., Delvau N., Burillo-Putze G., De Iaco F., Geldner G., Holmstrom P., Lambert Y., Sabbe M. (2013). Cyanide poisoning by fire smoke inhalation: A European expert consensus. Eur. J. Emerg. Med..

[24] Huzar T.F., George T., Cross J.M. (2013). Carbon monoxide and cyanide toxicity: Etiology, pathophysiology and treatment in inhalation injury. Expert Rev. Respir. Med..

[25] Guzman J.A. (2012). Carbon monoxide poisoning. Crit. Care Clin..

[26] Coburn R.F., Blakemore W.S., Forster R.E. (1963). Endogenous carbon monoxide production in man. J. Clin. Investig..

[27] Sjöstrand T. (1949). Endogenous formation of carbon monoxide in man under normal and pathological conditions. Scand. J. Clin. Lab. Investig..

[28] Sjöstrand T. (1951). Endogenous formation of carbon monoxide. the co concentration in the inspired and expired air of hospital patients. Acta Physiol. Scand..

[29] Baranano D.E., Ferris C.D., Snyder S.H. (2001). Atypical neural messengers. Trends Neurosci..

[30] Eichhorn L., Thudium M., Jüttner B. (2018). The Diagnosis and Treatment of Carbon Monoxide Poisoning. Dtsch. Arztebl. Int..

[31] Widdop B. (2002). Analysis of carbon monoxide. Ann. Clin. Biochem..

[32] Nager E.C., O’Connor R.E. (1998). Carbon monoxide poisoning from spray paint inhalation. Acad. Emerg. Med..

[33] Zegdi R., Perrin D., Burdin M., Boiteau R., Tenaillon A. (2002). Increased endogenous carbon monoxide production in severe sepsis. Intensiv. Care Med..

[34] Marks G.S., Vreman H.J., McLaughlin B.E., Brien J.F., Nakatsu K. (2002). Measurement of endogenous carbon monoxide formation in biological systems. Antioxid. Redox Signal..

[35] Ahmed H., McLaughlin B.E., Soong J., Marks G.S., Brien J.F., Nakatsu K. (2005). The source of endogenous carbon monoxide formation in human placental chorionic villi. Cell Mol. Biol..

[36] McLaughlin B.E., Lash G.E., Graham C.H., Smith G.N., Vreman H.J., Stevenson D.K., Marks G.S., Nakatsu K., Brien J.F. (2001). Endogenous carbon monoxide formation by chorionic villi of term human placenta. Placenta.

[37] Fenn W.O. (1970). The burning of CO in tissues. Ann. N. Y. Acad. Sci..

[38] Peterson J.E., Stewart R.D. (1970). Absorption and elimination of carbon monoxide by inactive young man. Arch. Environ. Health.

[39] Schmitt F.O., Scott M.G. (1934). The effect of CO on tissue respiration. Am. J. Physiol..

[40] Nañagas K.A., Penfound S.J., Kao L.W. (2022). Carbon Monoxide Toxicity. Emerg. Med. Clin. N. Am..

[41] Kao L.W., Nañagas K.A. (2006). Toxicity associated with carbon monoxide. Clin. Lab. Med..

[42] Raub J.A., Mathieu-Nolf M., Hampson N.B., Thom S.R. (2000). Carbon monoxide poisoning—A public health perspective. Toxicology.

[43] Boehning D., Moon C., Sharma S., Hurt K., Hester L.D., Ronnett G.V., Shugar D., Snyder S.H. (2003). Carbon monoxide neurotransmission activated by Ck2 phosphorylation of heme oxygenase-2. Neuron.

[44] Mannaioni P.F., Vannacci A., Masini E. (2006). Carbon monoxide: The bad and the good side of the coin, from neuronal death to anti-inflammatory activity. Inflamm. Res..

[45] Zhang X., Shan P., Otterbein L.E., Alam J., Flavell R.A., Davis R.J., Choi A.M.K., Lee P.J. (2003). Carbon monoxide inhibition of apoptosis during ischemia-reperfusion lung injury is dependent on the p38 mitogen-activated protein kinase pathway and involves caspase 3. J. Biol. Chem..

[46] Taillé C., Almolki A., Benhamed M., Zedda C., Mégret J., Berger P., Lesèche G., Fadel E., Yamaguchi T., Marthan R. (2003). Heme oxygenase inhibits human airway smooth muscle proliferation via a bilirubin-dependent modulation of erk1/2 phosphorylation. J. Biol. Chem..

[47] Douglas C.G., Haldane J.S., Haldane J.B. (1912). The laws of combination of hæmoglobin with carbon monoxide and oxygen. J. Physiol..

[48] Haldane J.S. (1919). A Lecture on the Symptoms, Causes, and Prevention of Anoxaemia (Insufficient Supply of Oxygen to the Tissues), and the Value of Oxygen in its Treatment. Br. Med. J..

[49] Haldane J. (1900). The supposed oxidation of carbonic oxide in the living body. J. Physiol..

[50] Smith J.L. (1899). The Pathology of Gas Poisoning, Illustrated by Five Recent Cases. Br. Med. J..

[51] Haldane J. (1972). Medicolegal contributions of historical interest. The action of carbonic oxide on man. Forensic Sci..

[52] Llano A.L., Raffin T.A. (1990). Management of carbon monoxide poisoning. Chest.

[53] Sendroy J., Liu S.H., Van Slyke D.O. (1929). The gasometric estimation of the relative affinity constant for carbon monoxide and oxygen in whole blood at 38C. Am. J. Physiol..

[54] Roughton F.J.W., Darling R.C., Crocker G.H., Toth B., Jones J.H., Agostoni P., Swenson E.R., Bussotti M., Revera M., Meriggi P. (1944). The effect of carbon monoxide on the oxyhemoglobin dissociation curve. Am. J. Physiol. Content.

[55] Sladen R.N. (1981). The oxyhemoglobin dissociation curve. Int. Anesthesiol. Clin..

[56] Orellano T., Dergal E., Alijani M., Briggs C., Vasquez J., Goldbaum L.R., Absolon K.B. (1976). Studies on the mechanism of carbon monoxide toxicity. J. Surg. Res..

[57] Goldbaum L.R., Ramirez R.G., Absalon K.B. (1975). What is the mechanism of carbon monoxide toxicity?. Aviat. Space Environ. Med..

[58] Alonso J.R., Cardellach F., López S., Casademont J., Miró Ò. (2003). Carbon monoxide specifically inhibits cytochrome c oxidase of human mitochondrial respiratory chain. Pharmacol. Toxicol..

[59] Piantadosi C.A., Zhang J., Levin E.D., Folz R.J., Schmechel D.E. (1997). Apoptosis and delayed neuronal damage after carbon monoxide poisoning in the rat. Exp. Neurol..

[60] Thom S.R., Bhopale V.M., Han S.T., Clark J.M., Hardy K.R. (2006). Intravascular neutrophil activation due to carbon monoxide poisoning. Am. J. Respir. Crit. Care Med..

[61] Chin B.Y., Jiang G., Wegiel B., Wang H.J., MacDonald T., Zhang X.C., Gallo D., Cszimadia E., Bach F.H., Lee P.J. (2007). Hypoxia-inducible factor 1α stabilization by carbon monoxide results in cytoprotective preconditioning. Proc. Natl. Acad. Sci. USA.

[62] Thorn S.R., Keim L.W. (1989). Carbon monoxide poisoning: A Review epidemiology, pathophysiology, clinical findings, and treatment options including hyperbaric oxygen therapy. J. Toxicol. Clin. Toxicol..

[63] Cronje F.J., Carraway M.S., Freiberger J.J., Suliman H.B., Piantadosi C.A. (2004). Carbon monoxide actuates O2-limited heme degradation in the rat brain. Free Radic. Biol. Med..

[64] Piantadosi C.A., Penney D.G. (1996). Toxicity of carbon monoxide: Hemoglobin vs. histotoxic mechanisms. Carbon Monoxide.

[65] Goldbaum L.R., Orellano T., Dergal E. (1976). Mechanism of the toxic action of carbon monoxide. Ann. Clin. Lab. Sci..

[66] Norkool D.M., Kirkpatrick J.N. (1985). Treatment of acute carbon monoxide poisoning with hyperbaric oxygen: A review of 115 cases. Ann. Emerg. Med..

[67] Weaver L.K. (2009). Clinical practice. Carbon monoxide poisoning. N. Engl. J. Med..

[68] Myers R.A. (1984). Carbon monoxide poisoning. J. Emerg. Med..

[69] Brown S.D., Piantadosi C.A. (1992). Recovery of energy metabolism in rat brain after carbon monoxide hypoxia. J. Clin. Investig..

[70] Rottman S.J. (1991). Carbon monoxide screening in the ED. Am. J. Emerg. Med..

[71] Hill B.C. (1994). The pathway of CO binding to cytochrome *c* oxidase Can the gateway be closed?. FEBS Lett..

[72] Chance B., Erecinska M., Wagner M. (1970). Mitochondrial responses to carbon monoxide toxicity. Ann. N. Y. Acad. Sci..

[73] Hardy K.R., Thom S.R. (1994). Pathophysiology and treatment of carbon monoxide poisoning. J. Toxicol. Clin. Toxicol..

[74] Thom S., Ohnishi S., Ischiropoulos H. (1994). Nitric oxide released by platelets inhibits neutrophil b2 integrin function following acute carbon monoxide poisoning. Toxicol. Appl. Pharmacol..

[75] Florkowski C.M., Rossi M.L., Carey M.P., Poulton K., Dickson G.R., Ferner R.E. (1992). Rhabdomyolysis and acute renal failure following carbon monoxide poisoning: Two case reports with muscle histopathology and enzyme activities. J. Toxicol. Clin. Toxicol..

[76] Abdul-Ghaffar N.U., Farghaly M.M., Swamy A.S. (1996). Acute renal failure, compartment syndrome, and systemic capillary leak syndrome complicating carbon monoxide poisoning. J. Toxicol. Clin. Toxicol..

[77] Olson K.R. (1984). Carbon monoxide poisoning: Mechanisms, presentation, and controversies in management. J. Emerg. Med..

[78] DeBias D.A., Banerjee C.M., Birkhead N.C., Greene C.H., Scott S.D., Harrer W.V. (1976). Effects of carbon monoxide inhalation on ventricular fibrillation. Arch. Environ. Health Int. J..

[79] Sangalli B.C., Bidanset J.H. (1990). A review of carboxymyoglobin formation: A major mechanism of carbon monoxide toxicity. Vet. Hum. Toxicol..

[80] Verma A., Hirsch D.J., Glatt C.E., Ronnett G.V., Snyder S.H. (1993). Carbon monoxide: A putative neural messenger. Science.

[81] Coceani F. (1993). Carbon monoxide and dilation of blood vessels. Science.

[82] Barinaga M. (1993). Carbon monoxide: Killer to brain messenger in one step. Science.

[83] Fichtner A., Eichhorn L. (2022). Kohlenmonoxidintoxikation—Neue Aspekte und aktuelle leitlinienbasierte Empfehlungen [Carbon monoxide intoxication-New aspects and current guideline-based recommendations]. Anaesthesiologie.

[84] Butera R., Candura S.M., Locatelli C., Varango C., Li B., Manzo L. (1995). Neurological sequelae of carbon monoxide poisoning: Role of hyperbaric oxigen. Indorr. Environ..

[85] Yang Z.D. (1986). Observation of hyperbaric oxigen in 160 patients with later manifestations after acute carbon monoxide poisoning. J. Hyperbar. Med..

[86] Stewart R.D., Peterson J.E., Baretta E.D., Bachand R.T., Hosko M.J., Herrmann A.A. (1970). Experimental human exposure to carbon monoxide. Arch. Environ. Health Int. J..

[87] Stewart R.D., Peterson J.E., Fisher T.N., Hosko M.J., Baretta E.D., Dodd H.C., Herrmann A.A. (1973). Experimental human exposure to high concentrations of carbon monoxide. Arch. Environ. Health Int. J..

[88] Herman L.Y. (1998). Carbon monoxide poisoning presenting as an isolated seizure. J. Emerg. Med..

[89] Mori T., Nagai K. (2000). Carbon-monoxide poisoning presenting as an afebrile seizure. Pediatr. Neurol..

[90] Jones J.S., Lagasse J., Zimmerman G. (1994). Computed tomographic findings after acute carbon monoxide poisoning. Am. J. Emerg. Med..

[91] Miura T., Mitomo M., Kawai R., Harada K. (1985). CT of the brain in acute carbon monoxide intoxication: Characteristic features and prognosis. AJNR Am. J. Neuroradiol..

[92] Sawada Y., Takahashi M., Ohashi N., Fusamoto H., Maemura K., Kobayashi H., Yoshioka T., Sugimoto T. (1980). Computerised tomography as an indication of long-term outcome after acute carbon monoxide poisoning. Lancet.

[93] Okeda R., Funata N., Takano T., Miyazaki Y., Higashino F., Yokoyama K., Manabe M. (1981). The pathogenesis of carbon monoxide encephalopathy in the acute phase? Physiological and morphological correlation. Acta Neuropathol..

[94] Ginsberg M.D., Myers R.E. (1974). Experimental carbon monoxide encephalopathy in the primate I. Physiologic and metabolic aspects. Arch. Neurol..

[95] Ginsberg M.D., Myers R.E., McDonagh B.F. (1974). Experimental Carbon Monoxide Encephalopathy in the Primate. II. Clinical aspects, neuropathology, and physiologic correlation. Arch. Neurol..

[96] Penney D.G. (1988). Hemodynamic response to carbon monoxide. Environ. Health Perspect..

[97] Penney D.G. (1990). Acute carbon monoxide poisoning: Animal models: A review. Toxicology.

[98] Yanir Y., Shupak A., Abramovich A., Reisner S.A., Lorber A. (2002). Cardiogenic shock complicating acute carbon monoxide poisoning despite neurologic and metabolic recovery. Ann. Emerg. Med..

[99] Atkins E.H., Baker E.L. (1985). Exacerbation of coronary artery disease by occupational carbon monoxide exposure: A report of two fatalities and a review of the literature. Am. J. Ind. Med..

[100] Sheps D.S., Herbst M.C., Hinderliter A.L., Adams K.F., Ekelund L.G., O’Neil J.J., Goldstein G.M., Bromberg P.A., Dalton J.L., Ballenger M.N. (1990). Production of arrhythmias by elevated carboxyhemoglobin in patients with coronary artery disease. Ann. Intern. Med..

[101] Wolff E. (1994). Carbon monoxide poisoning with severe myonecrosis and acute renal failure. Am. J. Emerg. Med..

[102] Herman G.D., Shapiro A.B., Leikin J. (1988). Myonecrosis in carbon monoxide poisoning. Vet. Hum. Toxicol..

[103] Thom S.R. (1989). Smoke inhalation. Emerg. Med. Clin. N. Am..

[104] Krantz T., Thisted B., Strom J., Sørensen M.B. (1988). Acute carbon monoxide poisoning. Acta Anaesthesiol. Scand..

[105] Hosko M.J. (1970). The effect of carbon monoxide on the visual evoked response in man and the spontaneous electroencephalogram. Arch. Environ. Health Int. J..

[106] Hampson N.B., Piantadosi C.A., Thom S.R., Weaver L.K. (2012). Practice recommendations in the diagnosis, management, and prevention of carbone monoxide poisoning. Am. J. Resp. Crit. Care Med..

[107] Locatelli C., Casagranda I., Coen D., Demattè P., Demicheli V., Perraro F., Pesenti Campagnoni M., Porro F., Re G., Butera R. (2001). Linee Guida per la Gestione ed il Trattamento del Paziente con Intossicazione Cuta da Monossido di Carbonio.

[108] Longo L.D., Hill E.P. (1977). Carbon monoxide uptake and elimination in fetal and maternal sheep. Am. J. Physiol..

[109] Norman C.A., Halton D.M. (1990). Is carbon monoxide a workplace teratogen? A review and evaluation of the literature. Ann. Occup. Hyg..

[110] Koren G., Sharav T., Pastuszak A., Garrettson L.K., Hill K., Samson I., Rorem M., King A., Dolgin J.E. (1991). A multicenter, prospective study of fetal outcome following accidental carbon monoxide poisoning in pregnancy. Reprod. Toxicol..

[111] Cramer C.R. (1982). Fetal death due to accidental maternal carbon monoxide poisoning. J. Toxicol. Clin. Toxicol..

[112] Woody R.C., Brewster M.A. (1990). Telencephalic dysgenesis associated with presumptive maternal carbon monoxide intoxication in the first trimester of pregnancy. J. Toxicol. Clin. Toxicol..

[113] Caravati E.M., Adams C.J., Joyce S.M., Schafer N.C. (1988). Fetal toxicity associated with maternal carbon monoxide poisoning. Ann. Emerg. Med..

[114] Longo L.D. (1970). Carbon monoxide in the pregnant mother and fetus and its exchange across the placenta. Ann. N. Y. Acad. Sci..

[115] Vreman H.J., Mahoney J.J., Stevenson D.K. (1995). Carbon monoxide and carboxyhemoglobin. Adv. Pediatr..

[116] Takeuchi M., Abe Y. (2006). Carbon monoxide poisoning of children. Chudoku Kenkyu.

[117] Foster M., Goodwin S.R., Williams C., Loeffler J. (1999). Recurrent acute life-threatening events and lactic acidosis caused by chronic carbon monoxide poisoning in an infant. Pediatrics.

[118] Zworth M., Kareemi H., Boroumand S., Sikora L., Stiell I., Yadav K. (2023). Machine learning for the diagnosis of acute coronary syndrome using a 12-lead ECG: A systematic review. CJEM.

[119] Broderick J.P., Silva G.S., Selim M., Kasner S.E., Aziz Y., Sutherland J., Jauch E.C., Adeoye O.M., Hill M.D., Mistry E.A. (2023). Enhancing Enrollment in Acute Stroke Trials: Current State and Consensus Recommendations. Stroke.

[120] Savioli G., Ceresa I.F., Luzzi S., Lucifero A.G., Cambiè G., Manzoni F., Preda L., Ricevuti G., Bressan M.A. (2021). Mild Head Trauma (MHT) and Antiplatelet Therapy. Reply to Lorenzati et al. Comment on “Savioli et al. Mild Head Trauma: Is Antiplatelet Therapy a Risk Factor for Hemorrhagic Complications?”. Medicina.

[121] Savioli G., Ceresa I.F., Ciceri L., Sciutti F., Belliato M., Iotti G.A., Luzzi S., Del Maestro M., Mezzini G., Lafe E. (2020). Mild head trauma in elderly patients: Experience of an emergency department. Heliyon.

[122] Savioli G., Ceresa I., Macedonio S., Gerosa S., Belliato M., Luzzi S., Lucifero A., Manzoni F., Ricevuti G., Bressan M. (2021). Major Trauma in Elderly Patients: Worse Mortality and Outcomes in an Italian Trauma Center. J. Emerg. Trauma Shock.

[123] Savioli G., Ceresa I.F., Giordano M., Ferrari I., Varesi A., Floris V., Esposito C., Croesi B., Ricevuti G., Calvi M. (2021). The Reliability of Anamnestic Data in the Management of Clostridium Tetani Infection in Elderly. Front. Med..

[124] Lapenna R., Faralli M., Del Zompo M.R., Cipriani L., Mobaraki P.D., Ricci G. (2015). Reliability of an anamnestic questionnaire for the diagnosis of benign paroxysmal positional vertigo in the elderly. Aging Clin. Exp. Res..

[125] Aboraya A., Rankin E., France C., El-Missiry A., John C. (2006). The reliability of psychiatric diagnosis revisited: The clinician’s guide to improve the reliability of psychiatric diagnosis. Psychiatry.

[126] Lindner T., Slagman A., Senkin A., Möckel M., Searle J. (2015). Medical History of Elderly Patients in the Emergency Setting: Not an Easy Point-of-Care Diagnostic Marker. Emerg. Med. Int..

[127] Kennedy M., Hwang U., Han J.H. (2020). Delirium in the emergency department: Moving from tool-based research to system-wide change. J. Am. Geriatr. Soc..

[128] Carpenter C.R., Bassett E.R., Fischer G.M., Shirshekan J., Galvin J.E., Morris J.C. (2011). Four sensitive screening tools to detect cognitive dysfunction in geriatric emergency department patients: Brief Alzheimer’s screen, short blessed test, Ottawa 3DY, and the caregiver-completed AD8. Acad. Emerg. Med..

[129] Savioli G., Ceresa I., Guarnone R., Muzzi A., Novelli V., Ricevuti G., Iotti G., Bressan M., Oddone E. (2021). Impact of Coronavirus Disease 2019 Pandemic on Crowding: A Call to Action for Effective Solutions to “Access Block”. West J. Emerg. Med..

[130] Savioli G., Ceresa I.F., Novelli V., Ricevuti G., Bressan M.A., Oddone E. (2021). How the coronavirus disease 2019 pandemic changed the patterns of healthcare utilization by geriatric patients and the crowding: A call to action for effective solutions to the access block. Intern. Emerg. Med..

[131] Lauque D., Khalemsky A., Boudi Z., Östlundh L., Xu C., Alsabri M., Onyeji C., Cellini J., Intas G., Soni K.D. (2022). Length-of-Stay in the Emergency Department and In-Hospital Mortality: A Systematic Review and Meta-Analysis. J. Clin. Med..

[132] Wu L., Chen X., Khalemsky A., Li D., Zoubeidi T., Lauque D., Alsabri M., Boudi Z., Kumar V.A., Paxton J. (2023). The Association between Emergency Department Length of Stay and In-Hospital Mortality in Older Patients Using Machine Learning: An Observational Cohort Study. J. Clin. Med..

[133] Savioli G., Ceresa I.F., Bressan M.A., Piccini G.B., Varesi A., Novelli V., Muzzi A., Cutti S., Ricevuti G., Esposito C. (2023). Five Level Triage vs. Four Level Triage in a Quaternary Emergency Department: National Analysis on Waiting Time, Validity, and Crowding—The CREONTE (Crowding and RE-Organization National TriagE) Study Group. Medicina.

[134] Savioli G., Ceresa I.F., Maggioni P., Lava M., Ricevuti G., Manzoni F., Oddone E., Bressan M.A. (2020). Impact of ED Organization with a Holding Area and a Dedicated Team on the Adherence to International Guidelines for Patients with Acute Pulmonary Embolism: Experience of an Emergency Department Organized in Areas of Intensity of Care. Medicines.

[135] Savioli G., Ceresa I.F., Manzoni F., Ricevuti G., Bressan M.A., Oddone E. (2020). Role of a Brief Intensive Observation Area with a Dedicated Team of Doctors in the Management of Acute Heart Failure Patients: A Retrospective Observational Study. Medicina.

[136] Crocker P.J., Walker J.S. (1985). Pediatric carbon monoxide toxicity. J. Emerg. Med..

[137] Strope G.L., Watkins C.G. (1986). Chronic carbon monoxide poisoning as a major contributing factor in the sudden infant death syndrome. Am. J. Dis. Child..

[138] Wright J. (2002). Chronic and occult carbon monoxide poisoning: We don’t know what we’re missing. Emerg. Med. J..

[139] Gilbert G.J., Glaser G.H. (1959). Neurologic manifestations of chronic carbon monoxide poisoning. N. Engl. J. Med..

[140] Khan K., Sharief N. (1995). Chronic carbon monoxide poisoning in children. Acta Paediatr..

[141] Myers R.A.M., DeFazio A., Kelly M.P. (1998). Chronic carbon monoxide exposure: A clinical syndrome detected by neuropsychological tests. J. Clin. Psychol..

[142] Knobeloch L., Jackson R. (1999). Recognition of chronic carbon monoxide poisoning. WMJ.

[143] Seger D., Welch L. (1994). Carbon monoxide controversies: Neuropsychological tessting, mechanism of toxicity, and hyperbaric oxyge. Ann. Emerg. Med..

[144] Min S.K. (1986). A brain syndrome associated with delayed neuropsychiatric sequelae following acute carbon monoxide intoxication. Acta Psychiatr. Scand..

[145] Thom S.R., Taber R.L., I Mendiguren I., Clark J.M., Hardy K.R., Fisher A.B. (1995). Delayed neuropsychologic sequelae after carbon monoxide poisoning: Prevention by treatment with hyperbaric oxygen. Ann. Emerg. Med..

[146] Choi I.S. (1983). Delayed neurologic sequelae in carbon monoxide intoxication. Arch. Neurol..

[147] Smith J.S., Brandon S. (1973). Morbility from acute carbon monoxide poisoning at three-year follow-up. Br. Med..

[148] Mathieu D., Nolf M., Durocher A., Saulnier F., Frimat P., Furon D., Wattel F. (1985). Acute carbon monoxide poisoning risk of late sequelae and treatment by hyperbaric oxygen. J. Toxicol. Clin. Toxicol..

[149] Huang C.C., Chen T.H., Ho C.H., Chen Y.C., Hsu C.C., Lin H.J., Wang J.J., Chang C.P., Guo H.R. (2021). Increased risk of congestive heart failure following carbon monoxide poisoning. Circ. Heart Fail..

[150] Huang C.C., Ho C.H., Chen Y.C., Lin H.J., Hsu C.C., Wang J.J., Su S.B., Guo H.R. (2019). Risk of myocardial infarction after carbon monoxide poisoning: A nationwide population-based cohort study. Cardiovasc. Toxicol..

[151] Huang C.C., Ho C.H., Chen Y.C., Hsu C.C., Lin H.J., Wang J.J., Su S.B., Guo H.R. (2020). Effects of hyperbaric oxygen therapy on acute myocardial infarction following carbon monoxide poisoning. Cardiovasc. Toxicol..

[152] Huang C.C., Ho C.H., Chen Y.C., Hsu C.C., Lin H.J., Wang J.J., Su S.B., Guo H.R. (2022). Association between carbon monoxide poisoning and adrenal insufficiency: A nationwide cohort study. Sci. Rep..

[153] Huang C.C., Chung M.H., Weng S.F., Chien C.C., Lin S.J., Lin H.J., Guo H.R., Su S.B., Hsu C.C., Juan C.W. (2014). Long-Term prognosis of patients with carbon monoxide poisoning: A nationwide cohort study. PLoS ONE.

[154] Huang C.C., Ho C.H., Chen Y.C., Lin H.J., Hsu C.C., Wang J.J., Su S.B., Guo H.R. (2017). Hyperbaric oxygen therapy is associated with lower short- and long-term mortality in patients with carbon monoxide poisoning. Chest.

[155] Huang C.C., Ho C.H., Chen Y.C., Lin H.J., Hsu C.C., Wang J.J., Su S.B., Guo H.R. (2017). Increased risk for diabetes mellitus in patients with carbon monoxide poisoning. Oncotarget.

[156] Nieman L.K. (2019). Patient Education: Adrenal Insufciency (Addison’s Disease) (Beyond the Basics). https://medilib.ir/uptodate/show/15599.

[157] Bornstein S.R. (2009). Predisposing Factors for Adrenal Insufficiency. N. Engl. J. Med..

[158] Inoue O. (1985). Clinical description of dementia. Kangogaku Zasshi.

[159] Inoue S. (1986). Various types of dementia. (3). Subdural hematoma, hypothyroidism, anterior pituitary hypofunction, carbon monoxide poisoning, and pseudodementia. Kangogaku Zasshi.

[160] Huang C.C., Ho C.H., Chen Y.C., Hsu C.C., Lin H.J., Su S.B., Wang J.J., Guo H.R. (2019). Increased risk for hypothyroidism associated with carbon monoxide poisoning: A nationwide population-based cohort study. Sci. Rep..

[161] Didier K., Bolko L., Giusti D., Toquet S., Robbins A., Antonicelli F., Servettaz A. (2018). Autoantibodies Associated With Connective Tissue Diseases: What Meaning for Clinicians?. Front. Immunol..

[162] Kumagai S., Jikimoto T., Saegusa J. (2003). Pathological roles of oxidative stress in autoimmune diseases. Rinsho Byori.

[163] Huang C.C., Ho C.H., Chen Y.C., Hsu C.C., Lin H.J., Wang J.J., Guo H.R. (2020). Autoimmune Connective Tissue Disease Following Carbon Monoxide Poisoning: A Nationwide Population-Based Cohort Study. Clin. Epidemiol..

[164] Pagano G., Castello G., Pallardó F.V. (2013). Sjøgren’s syndrome-associated oxidative stress and mitochondrial dysfunction: Prospects for chemoprevention trials. Free. Radic. Res..

[165] Perl A. (2013). Oxidative stress in the pathology and treatment of systemic lupus erythematosus. Nat. Rev. Rheumatol..

[166] Cooper G.S., Dooley M.A., Treadwell E.L., St Clair E.W., Gilkeson G.S. (2002). Risk factors for development of systemic lupus erythematosus: Allergies, infections, and family history. J. Clin. Epidemiol..

[167] Van Hal T.W., van Bon L., Radstake T.R.D.J. (2011). A system out of breath: How hypoxia possibly contributes to the pathogenesis of systemic sclerosis. Int. J. Rheumatol..

[168] Hampson N.B., Mathieu D., Piantadosi C.A., Thom S.R., Weaver L.K. (2001). Carbon monoxide poisoning: Interpretation of randomized clinical trials and unresolved treatment issues. Undersea Hyperb. Med..

[169] Mastrangelo G., Mapp C., Crepet M. (1979). Ossido di carboniop. Medicina del Lavoro.

[170] Chiappino G., Tomasini M., Chiappino G., Tomasini M. (1994). Gas tossici. Medicina del Lavoro.

[171] Mennoia V., Gazzaruso C., Geroldi D., Cadura S.M. (1999). Fenotipi di apolipoproteina(a) come indicatori di suscettiblità genetica per rischio cardiovascolare in medicina del lavoro. G. Ital. Med. Lav. Erg..

[172] Barret L., Danel V., Faure J. (1985). Carbon monnoxide poisoning, a diagnosis frequently overlooked. J. Toxicol. Clin. Toxicol..

[173] Ernst A., Zibrak J.D. (1998). Carbon monoxide poisoning. N. Engl. J. Med..

[174] Weaver L.K., Howe S., Hopkins R., Chan K.J. (2000). Carboxyhemoglobin half-life in carbon monoxide-poisoned patients treated with 100% oxygen at atmospheric pressure. Chest.

[175] Weaver L.K., Valentine K.J., Hopkins R.O. (2007). Carbon monoxide poisoning: Risk factors for cognitive sequelae and the role of hyperbaric oxygen. Am. J. Respir. Crit. Care Med..

[176] Touger M., Gallagher E., Tyrell J. (1995). Relationship between venous and arterial carboxyhemoglobin levels in patients with suspected carbon monoxide poisoning. Ann. Emerg. Med..

[177] Candura S.M., Fonte R., Finozzi E., Guarnone F., Taglione L., Manzo L., Biscaldi G. (1993). Inquinamento indoor: Presentazione di due casi clinici di ossicarbonismo occulto. G. Ital. Med. Lav..

[178] Winter P.M., Miller J.N. (1976). Carbon monoxide poisoning. JAMA.

[179] Pace N., Strajman E., Walker E.L. (1950). Acceleration of Carbon Monoxide Elimination in Man by High Pressure Oxygen. Science.

[180] Jay G.D., McKindley D.S. (1997). Alterations in pharmacokinetics of carboxyhemoglobin produced by oxygen under pressure. Undersea Hyperb. Med..

[181] Araki R., Nashimoto I., Takano T. (1988). The effect of hyperbaric oxygen on cerebral hemoglobin oxygenation and dissociation rate of carboxyhemoglobin in anesthetized rats: Spectroscopic approach. Adv. Exp. Med. Biol..

[182] Smith G. (1962). The treatment of carbon monoxide poisoning with oxygen at two atmospheres absolute. Ann. Occup. Hyg..

[183] Loyning Y. (1961). Treatment with oxygen under pressure in carbon monoxide poisoning. Tidsskr. Nor. Laegeforen..

[184] Thom S.R. (1990). Antagonism of carbon monoxide-mediated brain lipid peroxidation by hyperbaric oxygen. Toxicol. Appl. Pharmacol..

[185] Brown S.D., Piantadosi C.A. (1989). Reversal of carbon monoxide-cytochrome c oxidase binding by hyperbaric oxygen in vivo. Adv. Exp. Med. Biol..

[186] Thom S. (1993). Functional inhibition of leukocyte b2 integrins by hyperbaric oxygen in carbon monoxide-mediated brain injury in rats. Toxicol. Appl. Pharmacol..

[187] Jiang J., Tyssebotn I. (1997). Normobaric and hyperbaric oxygen treatment of acute carbon monoxide poisoning in rats. Undersea Hyperb. Med..

[188] Gesell L.B., Trott A. (2008). De novo cataract development following a standard course of hyperbaric oxygen therapy. Undersea Hyperb. Med..

[189] Gesell L.B. (2008). Hyperbaric Oxygen 2009: Indications and Results: The Hyperbaric Oxygen Therapy Committee Report.

[190] Weaver L.K., Hopkins R.O., Chan K.J., Churchill S., Elliott C.G., Clemmer T.P., Orme J.F., Thomas F.O., Morris A.H. (2002). Hyperbaric oxygen for acute carbon monoxide poisoning. N. Engl. J. Med..

[191] Ducassé J.L., Celsis P., Marc-Vergnes J.P. (1995). Non-comatose patients with acute carbon monoxide poisoning: Hyperbaric or normobaric oxygenation?. Undersea Hyperb. Med..

[192] Raphael J.C., Elkharrat D., Jars-Guincestre M.C., Chastang C., Vercken J.B., Chasles V., Gajdos P. (1989). Trial of normobaric and hyperbaric oxygen for acute carbon monoxide intoxication. Lancet.

